# Mechanistic Investigation of Cryptotanshinone and Curcumin Combination in Attenuating BVDV-1-Induced Injury in Bovine Ovarian Granulosa Cells

**DOI:** 10.3390/vetsci13101002

**Published:** 2026-09-23

**Authors:** Xiaoliang Chen, Kang Zhang, Haipeng Feng, Lei Wang, Jingyan Zhang, Xiaorong Lu, Guowei Xu, Siqi Liu, Xiaowei Feng, Junyan Wang, Jianxi Li

**Affiliations:** 1 Hebei Key Laboratory of Traditional Chinese Veterinary Medicine, Hebei Agricultural University, Baoding 071000, China; 20231200111@pgs.hebau.edu.cn; 2 Traditional Chinese Veterinary Technology Innovation Center of Gansu Province, Key Laboratory of Veterinary Pharmaceutical Development of Ministry of Agriculture and Rural Affairs of China, Lanzhou Institute of Husbandry and Pharmaceutical Sciences of Chinese Academy of Agricultural Sciences, Lanzhou 730050, China; zhangkang@caas.cn (K.Z.); fenghaipeng@caas.cn (H.F.); wanglei03@caas.cn (L.W.); zhangjingyan@caas.cn (J.Z.); luxr1993@163.com (X.L.); xuguowei@caas.cn (G.X.); 20251200187@pgs.hebau.edu.cn (S.L.); 82101231377@caas.cn (X.F.); 1073323140020@st.gsau.edu.cn (J.W.); 3 College of Veterinary Medicine, Gansu Agricultural University, Lanzhou 730070, China

**Keywords:** cryptotanshinone, curcumin, BVDV-1, BOGCs, network pharmacology, molecular docking, mechanism of action

## Abstract

Bovine viral diarrhea virus (BVDV) is a widespread pathogen causing significant economic losses in cattle. There is an urgent need for drugs to treat BVDV-induced reproductive dysfunction. This study investigated the mechanisms and targets of the cryptotanshinone–curcumin (CRY-CUR) combination against BVDV-1. Overlapping targets of CRY, CUR, and BVDV were screened using TCMSP, ChEMBL, and GeneCards databases. A Venn diagram was generated, followed by GO and KEGG enrichment analyses via DAVID. A PPI network was constructed using Cytoscape, and molecular docking was performed with AutoDock. Anti-BVDV-1 activity was verified in bovine ovarian granulosa cells (BOGCs) using RT-qPCR and immunofluorescence. A total of 108 overlapping targets were identified, with four core targets (STAT3, TNF, BCL-2, and EGFR) selected. Molecular docking showed strong binding affinities. Enrichment analysis indicated involvement in calcium ion binding, apoptosis, and TNF/NF-κB pathways. In vitro, the CRY-CUR combination suppressed BVDV-1 replication and reversed abnormal mRNA expression of the core targets. The CRY-CUR combination may inhibit BVDV-1 replication, potentially through modulation of BVDV-1-related targets such as STAT3, TNF, BCL-2, and EGFR and their associated signaling pathways.

## Simple Summary

Bovine viral diarrhea virus (BVDV) induces oophoritis and results in substantial economic losses to dairy cattle worldwide. Both cryptotanshinone and curcumin exhibit anti-inflammatory and antiviral properties. In this study, network pharmacology, molecular docking and in vitro experiments were combined to explore the mechanism by which the cryptotanshinone and curcumin combination alleviates BVDV-1-triggered damage in bovine ovarian granulosa cells. STAT3, TNF, BCL-2 and EGFR were identified as the core targets of the combination against BVDV-1. Molecular docking analysis revealed strong binding affinities. Enrichment analysis demonstrated that calcium ion binding, apoptosis and the TNF/NF-κB signaling pathways are the key functional modules involved. In vitro assays confirmed that the cryptotanshinone and curcumin combination suppresses BVDV-1 replication and reverses the aberrant mRNA expression of STAT3, TNF, BCL-2 and EGFR. Therefore, these findings demonstrate that the cryptotanshinone and curcumin combination exerts prominent inhibitory activity against BVDV-1, which is promising for the control of BVDV-1 infection.

## 1. Introduction

Bovine viral diarrhea virus (BVDV) is one of the major pathogens severely endangering the dairy cattle industry; it can infect a variety of domestic and wild animals, including sheep, goats, deer, and pigs [1]. In 2016, BVDV was estimated to impose nearly $2 billion in financial losses in the U.S. dairy cattle industry [2]. In addition to inducing bovine viral diarrhea-mucosal disease (BVD-MD), BVDV impairs reproductive performance and induces immunosuppression in dairy cattle, disrupts estrous cycles, triggers abortion and stillbirth in pregnant cows, and establishes persistent infection with lifelong immunodeficiency in calves [3]. These clinical manifestations pose a major challenge to the livestock industry worldwide. BVDV occurs in cytopathic (CP) and non-cytopathic (NCP) forms, which are distinguished by their different impacts on infected host cells. During long-term viral evolution, both CP BVDV and NCP BVDV have developed diverse mechanisms that enable them to circumvent host innate antiviral defenses. NCP BVDV is closely associated with persistent infection (PI) in cattle, and PI cattle serve as the main reservoir for BVDV transmission. Notably, genomic alterations of NCP BVDV in persistently infected animals can give rise to the CP biotype, which may subsequently lead to mucosal disease [4,5]. Although BVDV vaccines are commercially available, their efficacy in preventing infection and mitigating clinical disease remains suboptimal. Moreover, cattle diagnosed with persistent BVDV infection are typically culled upon clinical detection to prevent viral transmission within the herd. Therefore, it is essential to develop some safe and potent effective traditional Chinese herbal treatments for counteracting BVDV occurrence and infection.

Cryptotanshinone (CRY), a lipophilic diterpenoid constituent isolated from Salvia miltiorrhiza Bunge, has been extensively applied for the alleviation of menstrual disorders and the suppression of inflammatory reactions [6]. Previous research has demonstrated that CRY can ameliorate endometriosis, restore uterine endocrine imbalance and metabolic dysfunction in rats with polycystic ovary syndrome (PCOS), and exert prominent antiviral activity against severe acute respiratory syndrome coronavirus 2 (SARS-CoV-2) and porcine reproductive and respiratory syndrome virus (PRRSV) [7,8,9,10]. Furthermore, CRY modulates cytokine secretion and constrains BVDV replication via the negative regulation of cellular apoptosis [7]. Curcumin (CUR), a natural phenolic bioactive compound, is extracted from the rhizomes of Zingiberaceae species, predominantly Curcuma longa L. Previous research has demonstrated that curcumin harbors potent anti-inflammatory, anti-tumor and anti-polycystic ovary syndrome properties, and exerts broad-spectrum antiviral activity against multiple pathogens, including hepatitis B virus, human immunodeficiency virus type 1, porcine reproductive and respiratory syndrome virus and classical swine fever virus [8,9,10]. The inhibitory efficacy of curcumin (CUR) against BVDV infection has been robustly substantiated through an integrated approach encompassing molecular docking simulations, in vitro functional assays, and in vivo experimental validation [11,12,13,14]. Our previous study has demonstrated that CRY-CUR combination effectively alleviates BVDV-1-triggered inflammation, cellular apoptosis, and steroid hormone secretion disorders in bovine ovarian granulosa cells (BOGCs) [15]. Nevertheless, the mechanism underlying the inhibition of CRY-CUR combination on BVDV-1 replication remains unreported and poorly elucidated.

Network pharmacology provides a systematic approach to analyze, simulate and predict the potential therapeutic targets of traditional Chinese medicine (TCM) formulations. This methodology integrates the multi-component and multi-target features of TCM constituents with pathological mechanisms, thereby clarifying the complex interrelationships among drugs, target molecules and relevant diseases. This strategy has been extensively adopted in studies investigating numerous TCM preparations, which facilitates in-depth exploration of their clinical therapeutic value and pharmacological mechanisms [16,17].

This study aimed to evaluate the antiviral effects of the CRY-CUR combination against BVDV-1 in bovine ovarian granulosa cells (BOGCs) and to investigate its potential underlying mechanisms. Network pharmacology and molecular docking analyses were integrated with in vitro experiments to assess the effects of CRY-CUR on BVDV-1 infection and to identify potential molecular targets and signaling pathways involved in its antiviral activity.

## 2. Materials and Methods

### 2.1. Screening of Targets for CRY, CUR and BVDV

The chemical constituents of CRY and CUR were obtained from the Traditional Chinese Medicine Systems Pharmacology Database and Analysis Platform (TCMSP, https://old.tcmsp-e.com/tcmsp.php) (accessed 24 December 2025) and Chemical Biology and Medicinal Chemistry Database (chEMBL, https://www.ebi.ac.uk/chembl/) (accessed 24 December 2025), using the two medicines’ name as search keywords. After eliminating redundant entries, overlapping targets were extracted to obtain the core targets of CRY and CUR. The 3D structural files of CRY and CUR were separately acquired from the PubChem database (https://pubchem.ncbi.nlm.nih.gov/) (accessed 24 December 2025). The obtained potential targets were subsequently uploaded to the UniProt database (http://www.uniprot.org) (accessed 24 December 2025) for standardized protein target annotation, with Bos taurus set as the primary research species.

Target genes associated with BVDV were retrieved from the Genecards (https://www.genecards.org) (accessed 25 December 2025) and OMIM (https://www.omim.org) (accessed 25 December 2025) databases using relevant keyword, BVDV. All duplicate genes were subsequently eliminated to screen out the final BVDV-related target genes. First, the targets of CRY and CUR were integrated to obtain a combined dataset. The merged drug targets and BVDV-related targets were then imported to the Bioinformatics Analysis Tool (https://www.bioinformatics.com) (accessed 25 December 2025) for intersectant gene screening and Venn diagram construction. The overlapping hub targets were further visualized via Cytoscape 3.10.0 software (San Diego, CA, USA) to establish the corresponding interaction network.

### 2.2. GO and KEGG Enrichment Analysis

Core target genes were submitted to the DAVID database (https://davidbioinformatics.nih.gov) (accessed 27 December 2025) for functional enrichment analysis. GO and KEGG enrichment plots were performed using R 4.5.1 software (San Diego, CA, USA). For GO analysis, the top 10 terms in molecular function (MF), cellular component (CC), and biological process (BP) were selected for visualization. For KEGG analysis, the top 20 signaling pathways were selected for plotting.

### 2.3. Construction of the CRY-CUR Combination–BVDV Network

Cytoscape 3.10.0 software (San Diego, CA, USA) was used to analyze the CRY-CUR-BVDV-target interaction and construct the protein–protein interaction (PPI) network.

### 2.4. Molecular Docking

The downloaded 3D structures of CRY and CUR were optimized with AutoDock 1.5.6 (San Diego, CA, USA) and converted into PDBQT files. The top 4 targets ranked by degree were annotated in the UniProt database, and corresponding target protein structures were further retrieved from the PDB database utilizing their accession numbers. Ligand molecules were preprocessed via ADFR suite 1.0. Subsequently, global molecular docking was conducted between CRY and CUR and their corresponding target proteins by AutoDock Vina 1.2.5. The docking process was set to 32 processes with a grid spacing of 0.375, and all other parameters were set to default values. The docking scores generated by AutoDock Vina are negative, and a lower value indicates stronger binding affinity. A binding energy below −5 kcal/mol was considered to indicate moderate or high binding potential. Finally, the docking results were visualized using PyMOL 3.1.7.

### 2.5. Isolation of Bovine Ovarian Granulosa Cells

Ovarian specimens were collected from 20 healthy Holstein cows immediately after slaughter at a local abattoir in Wuzhong, Ningxia Hui Autonomous Region. Following excision, the ovaries were separately immersed in sterile physiological saline containing penicillin–streptomycin (100 IU/mL) (Gibco, Grand Island, NY, USA, 15140122), maintained at 30–37 °C during transportation, and delivered to the laboratory within 2 h of collection. According to the established protocol in our laboratory, healthy medium-sized follicles with a diameter of 5–8 mm were aspirated using a 5 mL syringe equipped with a 26G needle. The collected follicles were washed three times with PBS, followed by washing with DMEM/F12 (Gibco, Grand Island, NY, USA, C11330500BT). Bovine ovarian granulosa cells (BOGCs) were maintained within DMEM/F12 supplemented with 10% fetal bovine serum (FBS) (Gibco, Grand Island, NY, USA, A5669701) and 1% penicillin–streptomycin at 37 °C in 5% CO_2_ [18]. For each treatment condition, cells from the pooled population were seeded into three separate wells, which were considered technical replicates.

### 2.6. Identification of BOGCs

To identify whether the isolated cells were BOGCs, they were seeded onto confocal dishes and cultured until they reached 80% confluence. After washing with PBS, the cells were fixed with paraformaldehyde for 30 min and then rinsed again with PBS. Thereafter, samples were permeabilized with Triton X-100 (Beyotime, Shanghai, China, P0096) for 5 min and blocked with blocking buffer for 1 h, followed by additional PBS rinsing. Subsequently, the cells were reacted with primary antibodies against FSHR (1: 300) (Abcam, Shanghai, China, ab113421) and CYP19A1 (1: 200) (Abclonal, Wuhan, China, A12238) at 4 °C overnight. A negative control was included in parallel, in which the primary antibody was omitted [19]. After washing with PBS, goat anti-rabbit fluorescent secondary antibody (1:1000) (Proteinch, Wuhan, China, RGAR002) was added and the reaction proceeded for 1 h at ambient temperature while being protected from light. Finally, DAPI-containing antifade mounting medium (Beyotime, Shanghai, China, P0131) was dropped onto the slides, and immunofluorescence images were visualized and examined using a laser scanning confocal microscope.

### 2.7. Evaluation of the Effect of the CRY-CUR Combination on BVDV-1

CRY (20 μM) (China National Institutes for Drug Control, Beijing, China, Catalog No.: 110852-201807) and CUR (5 μM) (China National Institutes for Drug Control, Beijing, China, Catalog No.: 110823-202508) showed no cytotoxicity on BOGCs, and their combination exhibited antiviral activity consistent with previous descriptions [15]. Therefore, a CRY (20 μM)-CUR (5 μM) combination was selected for subsequent experiments. Madin–Darby bovine kidney (MDBK) cells and the BVDV NADL standard strain (cytopathic, CP type, CVCC AV67) were purchased from the China Institute of Veterinary Drug Control. The culture conditions for MDBK cells (China Institute of Veterinary Drug Control, Beijing, China, CVCC: CL21) were performed according to the method described in Reference [20]; the cells were cultured in RPMI 1640 medium (Gibco, Grand Island, NY, USA, 22400089) supplemented with 10% fetal bovine serum (FBS) and 1% penicillin/streptomycin at 37 °C in 5% CO_2_. Following BVDV infection, MDBK cells were cultured for 3–5 days. The infected cells and culture supernatants were then harvested and subjected to three freeze–thaw cycles, with freezing at −80 °C and thawing at room temperature. The lysates were centrifuged at 1000 rpm for 10 min to remove cellular debris, and the supernatants were collected as the viral stock. The viral titer was subsequently determined and was 10^−4.84^ TCID_50_/0.1 mL.

Prevention: To evaluate the preventive effect of CRY-CUR combination against BVDV-1, BOGCs were pretreated for 2 h using CRY-CUR combination. After discarding the supernatant, BOGCs were incubated with 100 TCID_50_ BVDV-1 at 37 °C for another 2 h. The virus-containing medium was then discarded and replaced with fresh complete medium, followed by a further 24 h of cultivation before sample harvesting.

Neutralizing: To assess whether pre-incubation with the CRY-CUR combination could reduce the ability of BVDV-1 to infect BOGCs, the CRY-CUR combination was mixed 1:1 (*v*/*v*) with BVDV-1 (100 TCID_50_), resulting in final concentrations of 10 μM CRY and 2.5 μM CUR. Uninfected cells treated with an equivalent volume of DMSO were used as the control. The mixture was incubated at 37 °C for 2 h and then added to BOGCs for 24 h before sample collection.

Therapeutics: To determine the therapeutic effect of CRY-CUR combination on BVDV-1 infection, BOGCs were infected with 100 TCID_50_ BVDV-1 at 37 °C for 2 h. After removing the supernatant, CRY-CUR combination was added with medium, and then the cells were incubated for 24 h prior to sample collection.

Adsorption: To investigate the inhibitory effect of CRY-CUR combination on the BVDV-1 adsorption stage, BOGCs were exposed to CRY-CUR combination at 4 °C for 2 h. After discarding the supernatant, the cells were infected with 100 TCID_50_ BVDV-1 and incubated at 37 °C for 2 h. The supernatant with the unabsorbed virus was removed, and the cells were cultured in complete medium at 37 °C for 24 h before sample collection.

Internalization: To explore the inhibitory effect of CRY-CUR combination on the BVDV-1 endocytosis stage, BOGCs were infected with 100 TCID_50_ BVDV-1 at 4 °C for 2 h. After removing the supernatant, CRY-CUR combination was added and the cells were incubated at 37 °C for 24 h, followed by sample collection.

### 2.8. Immunofluorescence

BOGCs treated with CRY-CUR combination or infected with BVDV-1 were washed with PBS. Uninfected BOGCs were processed in parallel under the same staining conditions and served as the negative control for BVDV infection. Cells were fixed with 4% paraformaldehyde at room temperature for 30 min. Subsequently, they were permeabilized with 0.1% Triton X-100 (Beyotime, Shanghai, China) for 10 min, and washed another three times with PBS for 5 min each, followed by incubation with BVDV FITC Conjugate (VMRD, Pullman, WA, USA; catalog No. P240301) overnight at 4 °C. After incubation, cells were washed once with PBS, mounted with antifade mounting medium containing DAPI, and observed under a laser scanning confocal microscope (LMS800, ZEISS, Oberkochen, Germany). Quantitative results were analyzed using Image J1.54p (National Institutes of Health, Bethesda, MD, USA) software.

### 2.9. BVDV-1 mRNA Quantitation

Samples were subjected to repeated freeze–thaw cycles, then supernatants were collected, and viral RNA was extracted using a Viral RNA/DNA Extraction Kit (Accurate Biotechnology Co., Ltd., Changsha, China, AG21034). Absolute quantification of viral RNA was performed by establishing a standard curve using recombinant plasmid standards. Primer pairs including the forward primer BVDV-F (5′-GGCATGCCCTTAGTAGGACT-3′) and reverse primer BVDV-R (5′-GCCATGTACAGCAGACAT-3′). BVDV RNA was subsequently quantified using a one-step probe-based reverse transcription quantitative PCR (RT-qPCR) assay (Accurate Biotechnology Co., Ltd., Changsha, China, AG11713). The real-time fluorescence quantitative reverse transcription PCR (RT-qPCR) program was set as follows: reverse transcription at 42 °C for 5 min, initial pre-denaturation at 95 °C for 30 s, followed by 40 cycles of denaturation at 95 °C for 5 s and annealing/extension at 60 °C for 30 s.

### 2.10. Relative Quantification of Gene Expression

Trizol reagent was utilized to extract total RNA from cultured cells. A NanoDrop ND-2000 spectrophotometer (Thermo Fisher Scientific, Waltham, MA, USA) was adopted to measure RNA purity and concentration. First-strand complementary DNA (cDNA) was reverse-transcribed from RNA templates using the cDNA synthesis kit from Accurate Biotechnology (Accurate Biotechnology Co., Ltd., Changsha, China, AG11728). Real-time fluorescent quantitative PCR was used to determine the expression levels of STAT3, EGFR, BCL-2 and TNF. Gene-specific primers were rationally designed using Primer-BLAST (BLAST+ 2.17.0), and all detailed primer sequences are summarized in Table 1. Subsequently, RT-qPCR amplification was carried out on either a LightCycler 480 real-time PCR system (Roche, Laval, QC, Canada) or an Applied Biosystems 7500 Real-Time PCR System (ABI, Los Angeles, CA, USA), with the SYBR^®^ Green Premix Pro Taq HS qPCR Kit containing Rox reference dye (Accurate Biotechnology Co., Ltd., Changsha, China, AG11718) adopted for fluorescent quantitative detection. The standardized thermal cycling protocol was set as follows: initial pre-denaturation at 95 °C for 30 s, followed by 40 cycles involving denaturation at 95 °C for 5 s and combined annealing and extension at 60 °C for 30 s. Upon completion of the amplification reaction, the relative mRNA expression levels of target genes were quantitatively calculated via the 2^−ΔΔCT^ comparative threshold cycle method [21]. All required primer pairs were professionally synthesized by Sangon Biotech Co., Ltd. (Shanghai, China).

### 2.11. Statistical Analysis

All experiments were performed in triplicate. Data were analyzed using IBM SPSS Statistics 26. Results were expressed as mean ± standard error of the mean (SEM). Statistical significance was determined by one-way analysis of variance (ANOVA). Figures were plotted using GraphPad Prism 9. Differences with *p* < 0.05 were regarded as statistically significant.

## 3. Results

### 3.1. CRY-CUR Combination and BVDV Targets

From the GeneCards and OMIM databases, A total of 17 and 64 CUR targets were retrieved from TCMSP and Chem databases, respectively, whereas 68 and 38 CRY targets were obtained from these two databases.. The union set of CRY and CUR targets was intersected with BVDV-related targets, yielding a total of 107 overlapping targets (Figure 1A). The target signaling network of CRY-CUR against BVDV was successfully constructed using Cytoscape_v3.10.4 software (Figure 1B). CRY-related targets included AR and CCND1, which are associated with hormone signaling and cell proliferation, respectively, as well as the pro-inflammatory cytokine TNF. CUR-related targets included the apoptosis regulator BCL2 and cell cycle-related CDKs. Several targets were shared by CRY and CUR, including EGFR, STAT3, and ESR1, which are involved in cell proliferation, immune-inflammatory responses, and hormone signaling (Figure 1B and Supplementary Table S1).

### 3.2. Functional Enrichment Analysis by KEGG and GO

GO enrichment analysis identified 99 biological process (BP), 30 cellular component (CC), and 59 molecular function (MF) terms at *p* < 0.05, and the top 10 terms in each category were visualized (Figure 2A). Among the BP terms, the targets were mainly enriched in the positive regulation of ERK1 and ERK2 cascade, positive regulation of interleukin-6 production, and positive regulation of calcium-mediated signaling. For CC, the targets were predominantly associated with the plasma membrane and cytoplasm. The major MF terms included nuclear receptor activity, protein homodimerization activity, and protein kinase binding (Figure 2A). KEGG enrichment analysis further revealed that the CRY-CUR-BVDV targets were associated with multiple signaling pathways, including the calcium, apoptosis, TNF, PI3K-Akt, NF-κB, Toll-like receptor, and HIF-1 signaling pathways (Figure 2B).

### 3.3. Protein–Protein Interaction Network Analysis

The 107 overlapping candidate genes between CRY-CUR-BVDV (Figure 1A) were imported into the STRING database to construct a protein–protein interaction (PPI) network. Based on degree values, the targets were classified into four groups: > 40, 20–40, 10–20, and <10. STAT3, TNF, BCL2, and EGFR exhibited degree values > 40 and were identified as the four core targets (Figure 3).

### 3.4. Molecular Docking Verification

This study integrated the results of network pharmacology, and Cytoscape was performed to verify the binding patterns between CRY, CUR and the four core targets of BVDV. Table 2 presents the docking binding energies and the number of hydrogen bonds for the interactions between CRY and CUR and four core targets. The binding energies of CRY and CUR to STAT3, TNF, BCL-2, and EGFR were all lower than −5 kcal/mol. All docking modes were further visualized. Specifically, CRY interacted with STAT3 at ARG335, with TNF at LEU103, with BCL-2 at LEU134, PHE150, GLU149, PHE101, ASP108, and MET112, and with EGFR at GLU1192, VAL726, and LEU718. CUR interacted with STAT3 at THR730 and LEU731, with TNF at GLN138, with BCL-2 at ASP100, and with EGFR at SER758 and ASN700 (Figure 4).

### 3.5. Identification of BOGCs

To identify whether the isolated cells were BOGCs, immunofluorescence staining was performed to detect the specific markers FSHR and CYP19A1. As shown in Figure 5, the cells exhibited strong positive signals for both FSHR and CYP19A1. These results confirmed that the isolated cells were BOGCs with high purity, displaying characteristic molecular markers.

### 3.6. Antiviral Effect of CRY-CUR Combination on BVDV-Infected BOGCs

To assess CRY-CUR’s antiviral function, we measured mRNA and protein expression levels of BVDV-1. As shown in Figure 6A–C, the BVDV-1 mRNA and protein levels in the infected group were significantly elevated as compared to the control (CON) group (*p* ˂ 0.05). Compared with the BVDV group, the prevention group reduced BVDV protein expression by approximately 30%, whereas the therapeutic and adsorption groups produced larger reductions of approximately 50%. The internalization treatment also decreased BVDV protein expression by approximately 39%. In addition, compared with BVDV-1-infected BOGCs, BOGCs treated with CRY-CUR combination prevention, therapeutic, adsorption, and internalization protocols exhibited significantly downregulation of mRNA and protein levels of BVDV-1 (*p* ˂ 0.05). Collectively, these finding indicated that the CRY-CUR combination suppress BVDV-1 replication.

### 3.7. Effect of the CRY-CUR Combination on the Expression of Target-Related Genes

Based on the molecular docking results in Section 3.4, STAT3, TNF, BCL2, and EGFR were selected for further experimental validation. As shown in Figure 7, compared with the CON group, the mRNA expression levels of EGFR, STAT3, and TNF were significantly increased, whereas BCL-2 mRNA expression was significantly decreased in the BVDV-1-infected group (*p* < 0.05). Compared with the BVDV-1-infected group, CRY-CUR treatment significantly downregulated the mRNA expression of EGFR, STAT3, and TNF (*p* < 0.01), while significantly increasing BCL-2 mRNA expression (*p* < 0.01).

## 4. Discussion

This study integrated network pharmacology, molecular docking, and in vitro cellular assays to systematically elucidate the underlying mechanism of the CRY-CUR combination against BVDV-1 infection. We found that the CRY-CUR combination can predict favorable interactions with core targets including TNF, STAT3, EGFR, and BCL-2. It may exert therapeutic effects by regulating calcium signaling, apoptosis, and NF-κB signaling pathways. By performing in vitro validation, we confirmed that the CRY-CUR combination exhibits inhibitory effects against BVDV-1 in BOGCs. Furthermore, CRY-CUR combination treatment reversed the aberrant expression of TNF, STAT3, EGFR, and BCL-2. Our findings highlight that the CRY-CUR combination is a potential candidate herbal medicine for intervention against BVDV-1 infection.

The core targets STAT3, TNF, BCL-2, and EGFR are closely associated with inflammatory responses, apoptosis, and the regulation of ovarian physiological functions. STAT3 serves as a pivotal signaling intermediary linking cytokine receptors to down-stream effector molecules, functioning both to attenuate tissue inflammatory damage and participate in the bidirectional regulation of viral replication [20,22]. STAT3 can inhibit apoptosis and protect cells from viral insult [7,23]. In the present study, BVDV-1 induced the elevation of STAT3, which in turn promoted viral replication, triggered apoptosis in BOGCs, and concurrently reduced BCL-2 expression. Notably, treatment with the CRY-CUR combination significantly alleviated the BVDV-1-induced upregulation of STAT3. These findings raise the possibility that BVDV-1 may modulate the intracellular environment of BOGCs through alterations in STAT3 signaling, potentially creating conditions favorable for viral transcription and replication [24].

TNF, a key pro-inflammatory cytokine, is highly expressed in BVDV-infected cattle and BOGCs [25]. Consistent with these documented observations, our results also demonstrated that BVDV-1 led to a marked increase in TNF, whereas intervention with CRY-CUR combination significantly counteracted this elevation. This finding implies that CRY-CUR attenuate BVDV-1-induced inflammatory responses in BOGCs via a repertoire of signaling cascades, potentially including the NF-κB pathway, the STAT3 signaling axis, and the calcium signaling pathway.

EGFR signaling is essential for maintaining normal follicular development, whereas its sustained aberrant upregulation disrupts follicular homeostasis. Clinical studies have reported elevated EGFR expression in ovarian granulosa cells isolated from patients with polycystic ovary syndrome [26]. BVDV-induced upregulation of EGFR activates the NF-κB pathway and promotes the release of TNF and HMGB1. In turn, HMGB1 further enhances EGFR levels [27], thereby establishing a vicious cycle. This cascade drives ovarian granulosa cell hyperproliferation, impairs oocyte meiotic maturation, and ultimately leads to ovulatory dysfunction, which may constitute a key mechanism underlying the reduced reproductive performance observed in BVDV-infected dairy cows [28]. In this study, treatment with the CRY-CUR combination significantly downregulated the abnormally elevated EGFR and reversed the BVDV-1-induced EGFR dysregulation. Nevertheless, whether the CRY–CUR combination can restore follicular developmental competence and mitigate ovarian functional deficits remains to be experimentally validated.

GO and KEGG pathway enrichment revealed that the activity of the CRY-CUR combination against BVDV is closely associated with multiple signaling pathways. Among these, the calcium signaling pathway exhibited the highest enrichment score, suggesting that it may serve as one of the crucial mechanisms underlying the antiviral action of this combination. Although a direct mechanistic link between the calcium signaling pathway and BVDV infection has not yet been established, accumulating evidence implicates this pathway in the replication of multiple RNA viruses, including pestiviruses. For instance, African swine fever virus (ASFV) promotes its own replication by mediating endoplasmic reticulum calcium release via the inositol trisphosphate receptor (IP3R) channel [29]; similarly, hepatitis C virus (HCV) activates intracellular calcium signaling, which in turn induces NLRP3 inflammasome activation in macrophages and triggers IL-1β secretion, culminating in a robust inflammatory response [30]. Accordingly, it is plausible that BVDV may elicit a strong inflammatory response in BOGCs through the calcium signaling pathway. It is well established that BVDV infection induces a pronounced inflammatory reaction. Previous studies have demonstrated that the cytopathic type of BVDV effectively activates the NLRP3 inflammasome, promoting the maturation and secretion of IL-1β [31]. Moreover, NF-κB, a core transcriptional complex that orchestrates inflammatory and immune responses, is significantly activated by cytopathic BVDV, leading to upregulated expression of NLRP3 inflammasome components and pro-IL-1β, ultimately exacerbating inflammatory injury [32]. In line with these observations, our KEGG analysis also enriched the NF-κB pathway, which suggested that CRY-CUR may suppress BVDV replication and inflammation through this pathway. This finding is supported by previous studies showing that various natural bioactive compounds can alleviate virus-induced inflammatory damage by targeting the NF-κB pathway [33,34]. However, additional functional assays are required to conclusively validate the modulatory effect of the CRY–CUR combination on NF-κB signaling activity.

BVDV can trigger ovarian inflammation, reduce reproductive efficiency in dairy cows, and even cause abortion and stillbirth [35]. Bovine granulosa cells play vital roles in ovarian development and dairy cow reproduction. Therefore, BOGCs were selected as an in vitro cellular model for functional verification in this study. Targeting viral entry into host cells represents a well-established and therapeutically promising strategy for the prevention and control of viral infections [36]. The results of this study show that viral mRNA and protein expression was not obviously inhibited in the virus neutralization test. These findings indicate that the primary antiviral effect of the CRY–CUR combination does not rely on directly neutralizing extracellular viral particles. Instead, it suppresses BVDV-1 infection by blocking viral adsorption and internalization into host cells as well as inhibiting viral replication.

To the best of our knowledge, this study represents the first application of network pharmacology to preliminarily identify the anti-BVDV potential of the CRY–CUR combination. However, functional validation such as pharmacological inhibition, genetic knockdown, and target overexpression was not performed. Moreover, while the CRY–CUR combination demonstrated inhibitory effects on BVDV-1 proliferation in vitro, in vivo efficacy remains unassessed due to the absence of animal experimentation. Therefore, further studies are warranted to comprehensively evaluate the therapeutic potential of this combination against BVDV infection.

## 5. Conclusions

In this study, network pharmacology and molecular docking were used to identify STAT3, TNF, EGFR, and BCL-2 as the core targets of the CRY-CUR combination. KEGG enrichment analysis revealed that the CRY-CUR combination is involved in the regulation of calcium, TNF, NF-κB, and apoptosis signaling pathway. Molecular docking results indicated that both CRY and CUR exhibited predicted favorable interactions with STAT, TNF, EGFR, and BCL-2. Collectively, the CRY-CUR combination is associated with multiple BVDV-1-related targets including STAT3, TNF, EGFR, and BCL-2, and may modulate host responses associated with inflammatory and apoptotic signaling pathways. However, these potential mechanisms require further validation through additional functional and mechanistic studies. Our findings suggest plausible mechanistic data for the anti-BVDV-1 activity of the CRY-CUR combination. This study provides a rational basis for the application of the CRY-CUR combination in the treatment of BVDV-1 and offers translational insights for the development and utilization of other natural medicines.

## Figures and Tables

**Figure 1:**
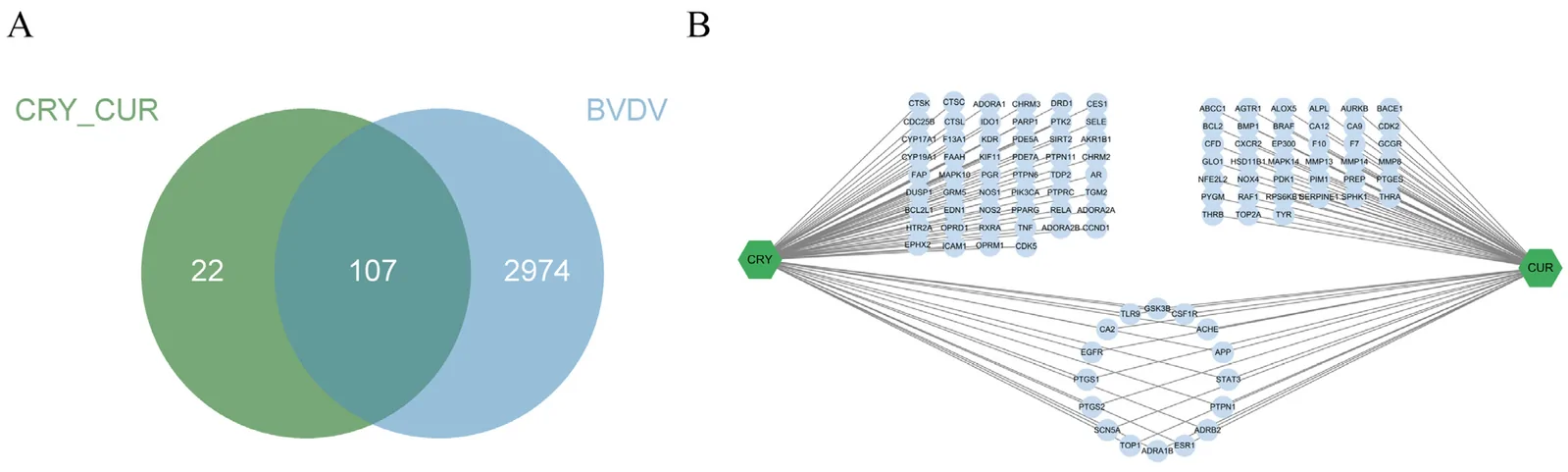
Target screening of CRY-CUR combination. (**A**) Venn diagram of overlapping targets between CRY, CUR and BVDV; (**B**) CRY-CUR combination target network diagram.

**Figure 2:**
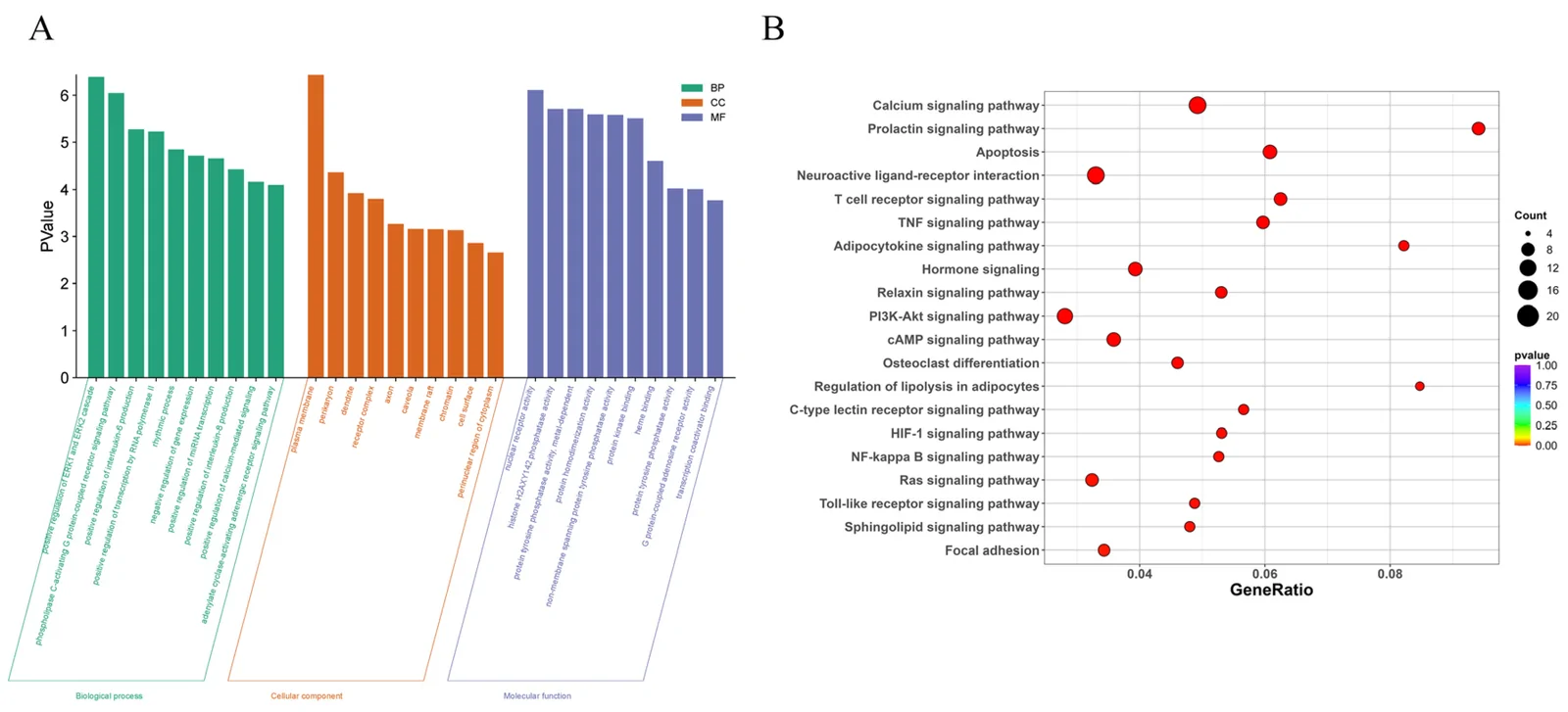
Results of KEGG and GO analysis of targets related to CRY-CUR combination. (**A**) Bar chart of KEGG analysis for targets related to CRY-CUR combination; (**B**) GO analysis results for targets related to CRY-CUR combination.

**Figure 3:**
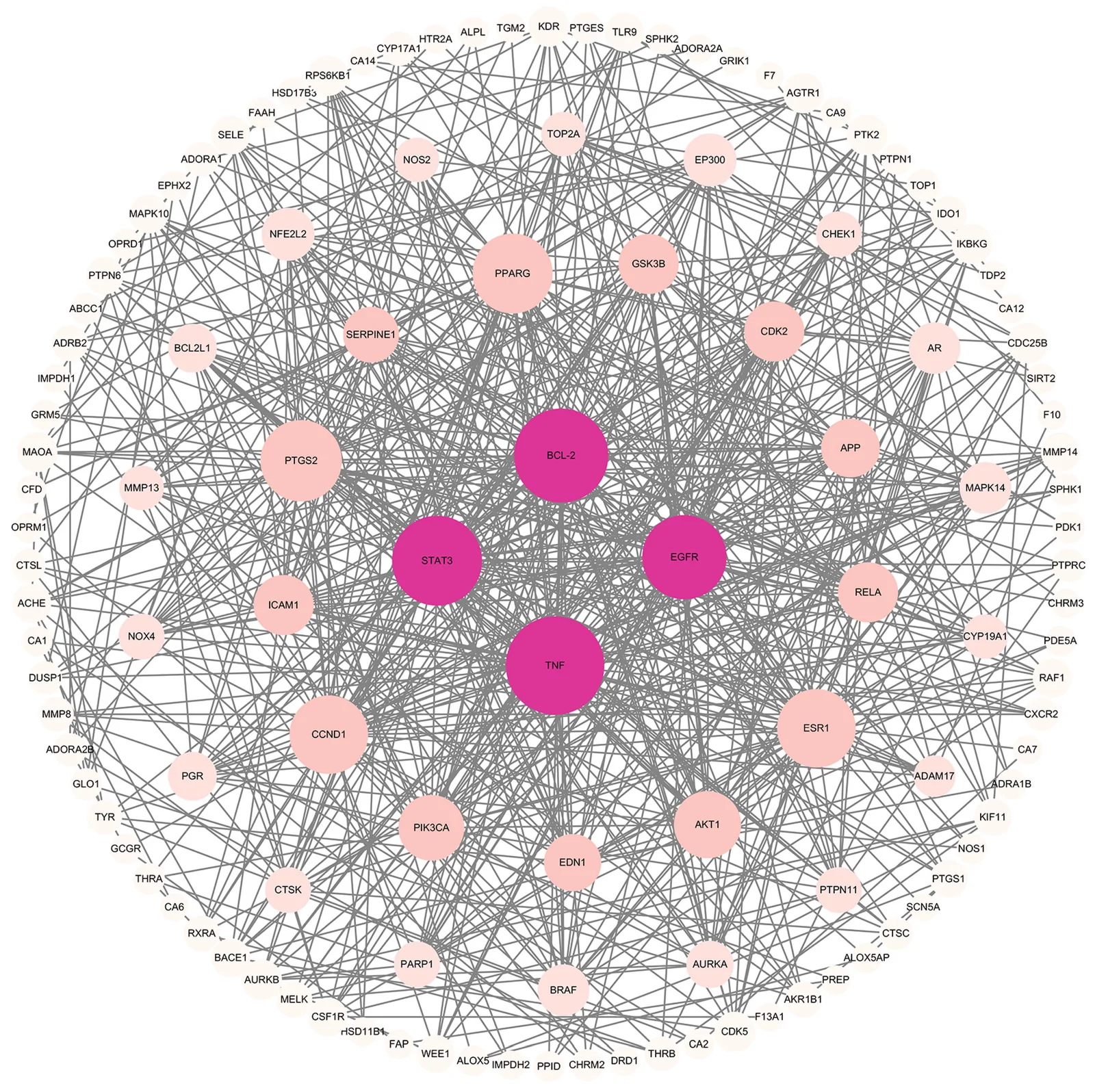
Protein–protein interaction (PPI) network analysis.

**Figure 4:**
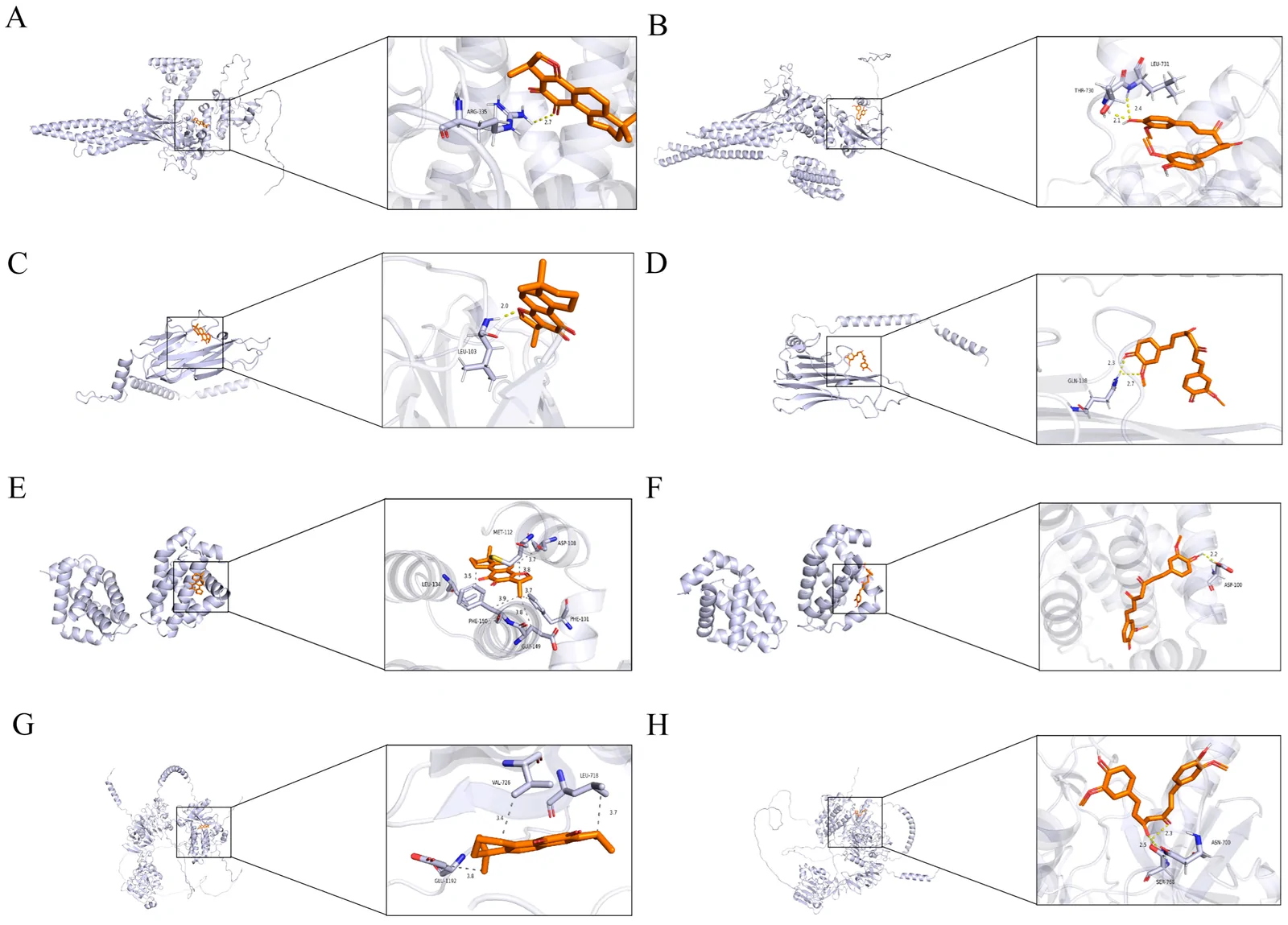
Molecular docking results of CRY and CUR with core targets. (**A**) Docking CRY and STAT3. (**B**) Docking CUR and STAT3. (**C**) Docking CRY and TNF. (**D**) Docking CUR and TNF. (**E**) Docking CRY and BCL-2. (**F**) Docking CUR and BCL-2. (**G**) Docking CRY and EGFR. (**H**) Docking CUR and EGFR.

**Figure 5:**
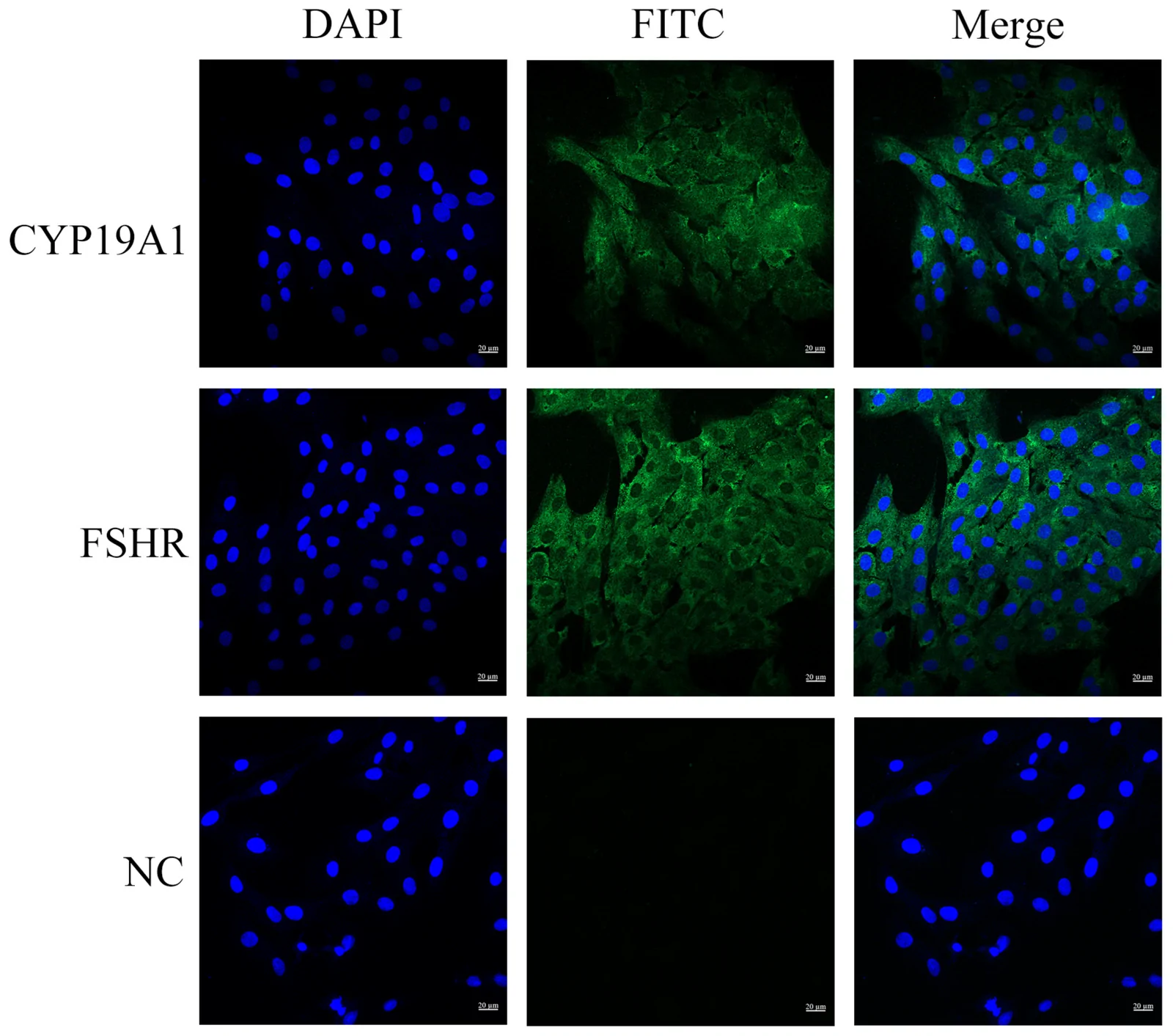
Immunofluorescence staining of BOGCs for CYP19A1 and FSHR.

**Figure 6:**
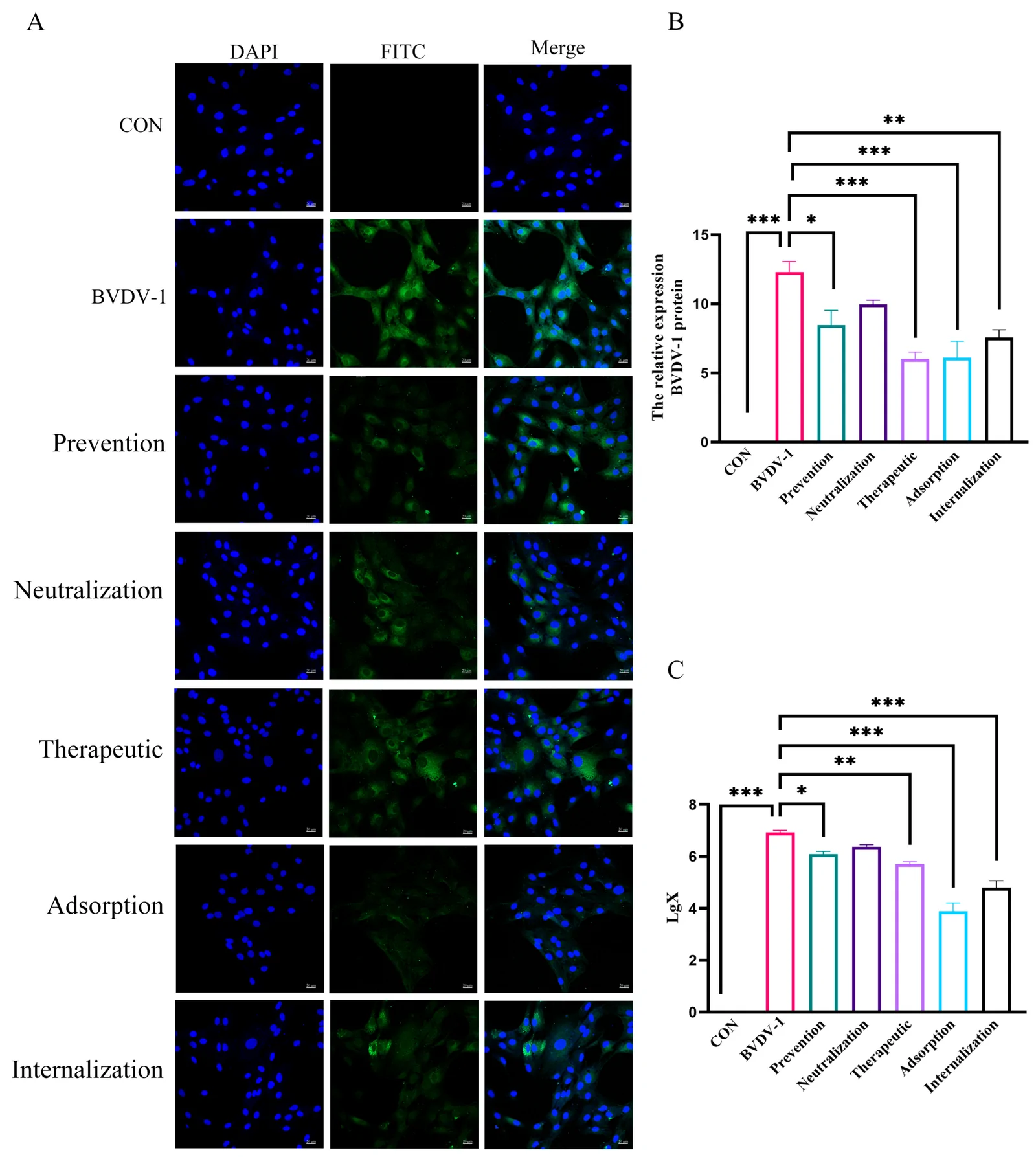
Anti-BVDV-1 effects of different intervention modes. (**A**) Immunofluorescence of BVDV-1 levels in each group. (**B**) As illustrated in (**A**), the Image J statistics of BVDV-1 protein levels. (**C**) BVDV-1 nucleic acid copy numbers. Data are presented as the mean ± *SEM* of three independent experiments (*n* = 3). The symbols *, ** and *** indicate significance levels of *p* < 0.05, *p* < 0.01 and *p* < 0.001, respectively.

**Figure 7:**
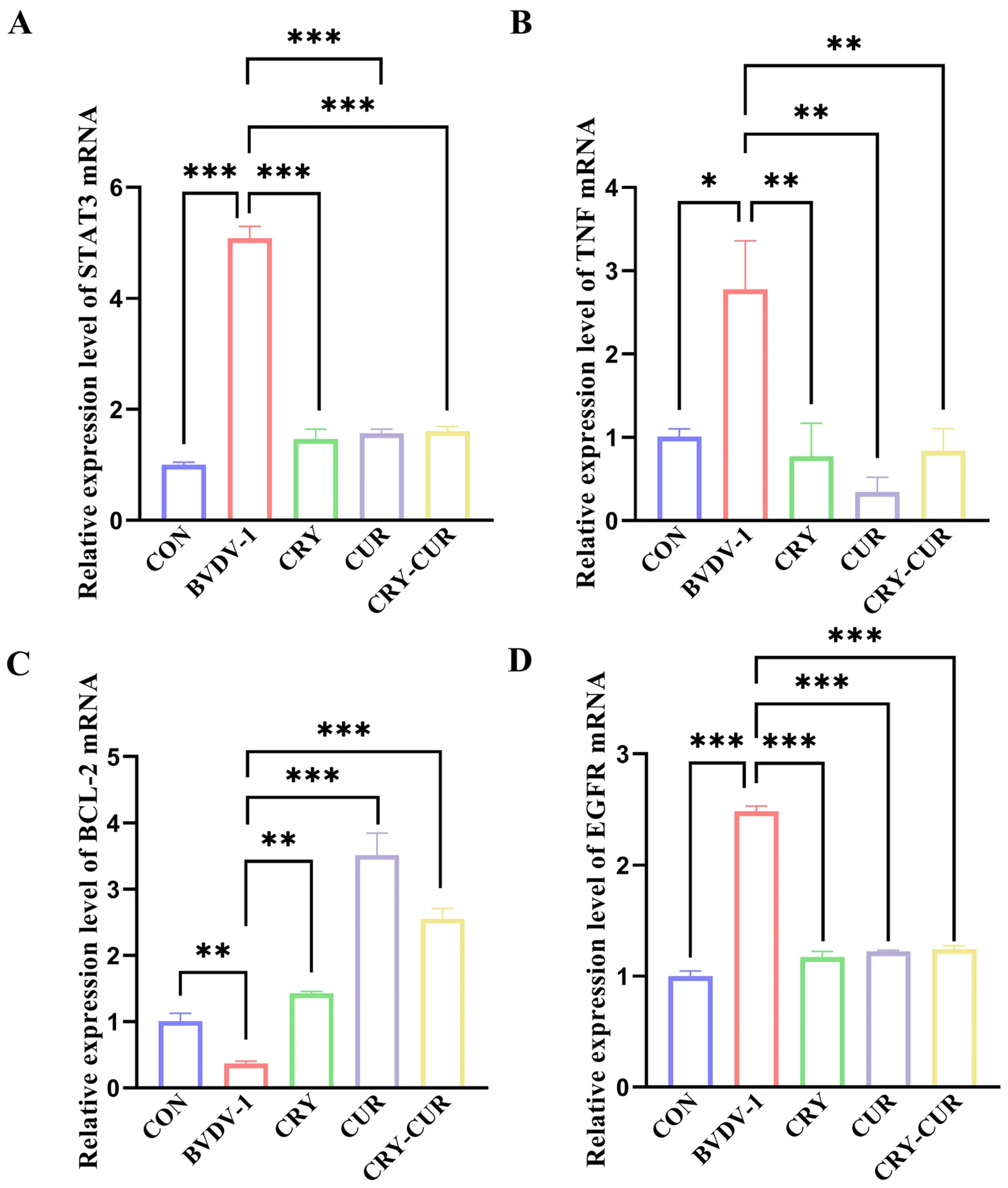
The CRY-CUR combination inhibits the targets of BVDV-1 on BOGCs. (**A**) The relative expression levels of STAT3 mRNA. (**B**) Relative mRNA expression of TNF. (**C**) The relative expression levels of BCL-2 mRNA. (**D**) Relative mRNA expression of EGFR. Data are presented as the mean ± SEM of three independent experiments (*n* = 3). * indicates *p* ˂ 0.05, ** indicates *p* ˂ 0.01, and *** indicates *p* < 0.001.

**Table 1:** Primers used in this study.

Gene	Sequence (5′-3′)	Product Length(bp)	Serial Number
STAT3	F: GCAATACCATTGACCTGCCG	118	XM_024979865.2
	R: TCGAACGTGAGGGACTCAAA		
EGFR	F: TCCTTCACACGTACTGCACC	263	XM_002696890.6
	R: GAGAAAACTGGCCATGCTG		
BCL-2	F: TCATGTGTGTGGAGAGCGTC	134	NM_001166486.1
	R: CTCCACAAAGGCGTCCCAG		
TNF	F: CCCACGTTGTAGCCGACAT	133	NM_173966.3
	R: ATGAGGTAAAGCCCGTCAGC		
β-actin	F: ATCGGCAATGAGCGGTTCC	143	NM_173979.3
	R: GTGTTGGCGTAGAGGTCCTTG		

**Table 2:** Molecular docking (binding energy) of molecules and core targets.

Core Target	CRY	CUR
STAT3	−8.097	−5.949
TNF	−6.601	−5.712
BCL-2	−7.979	−7.795
EGFR	−8.548	−6.504

Docking and binding affinity (Kcal/mol)/count of hydrogen bonds.

## Data Availability

The original contributions presented in this study are included in the article/Supplementary Material. Further inquiries can be directed to the corresponding author.

## Supplementary Materials

The following supporting information can be downloaded at: https://www.mdpi.com/article/10.3390/vetsci13101002/s1, Table S1: Number of Targets; Table S2: Targets.

## Abbreviations

The following abbreviations are used in this manuscript:

BVDV	bovine viral diarrhea virus
CRY	cryptotanshinone
CUR	curcumin
BOGCs	bovine ovarian granulosa cells
MDBK	Madin–Darby bovine kidney
MF	molecular function
CC	cellular component
BP	biological process
PPI	protein–protein interaction
FSHR	follicle-stimulating hormone receptor
CYP19A1	cytochrome P450 family 19 subfamily A member 1
